# Mapping the Parameter Space of tDCS and Cognitive Control via Manipulation of Current Polarity and Intensity

**DOI:** 10.3389/fnhum.2016.00665

**Published:** 2016-12-27

**Authors:** Elisabeth A. Karuza, Zuzanna Z. Balewski, Roy H. Hamilton, John D. Medaglia, Nathan Tardiff, Sharon L. Thompson-Schill

**Affiliations:** ^1^Department of Psychology, University of Pennsylvania, PhiladelphiaPA, USA; ^2^Department of Neurology, University of Pennsylvania, PhiladelphiaPA, USA

**Keywords:** tDCS, cognitive control, prefrontal cortex, Flanker task, neurostimulation

## Abstract

In the cognitive domain, enormous variation in methodological approach prompts questions about the generalizability of behavioral findings obtained from studies of transcranial direct current stimulation (tDCS). To determine the impact of common variations in approach, we systematically manipulated two key stimulation parameters—current polarity and intensity—and assessed their impact on a task of inhibitory control (the Eriksen Flanker). Ninety participants were randomly assigned to one of nine experimental groups: three stimulation conditions (anode, sham, cathode) crossed with three intensity levels (1.0, 1.5, 2.0 mA). As participants performed the Flanker task, stimulation was applied over left dorsolateral prefrontal cortex (DLPFC; electrode montage: F3-RSO). The behavioral impact of these manipulations was examined using mixed effects linear regression. Results indicate a significant effect of stimulation condition (current polarity) on the magnitude of the interference effect during the Flanker; however, this effect was specific to the comparison between anodal and sham stimulation. Inhibitory control was therefore improved by anodal stimulation over the DLPFC. In the present experimental context, no reliable effect of stimulation intensity was observed, and we found no evidence that inhibitory control was impeded by cathodal stimulation. Continued exploration of the stimulation parameter space, particularly with more robustly powered sample sizes, is essential to facilitating cross-study comparison and ultimately working toward a reliable model of tDCS effects.

## Introduction

With the recent surge in use of transcranial direct current stimulation (tDCS) has come a growing uncertainty about the reliability of this neuromodulatory technique. TDCS, a form of non-invasive electrical brain stimulation, hinges on a simple premise: *hypo-*polarization of a cortical area should increase neuronal excitability, while *hyper-*polarization should induce the opposite effect. Within the motor domain, this rationale has been largely supported at the neurophysiological level: when primary motor areas are hypo-polarized by positive current administered during anodal stimulation (A-tDCS), motor-evoked potentials (MEPs) recorded from peripheral muscles tend to increase in magnitude, indicating a boost in cortical excitability. In contrast, hyper-polarization of these areas via negative current administered during cathodal stimulation (C-tDCS) tends to diminish the amplitude of MEPs, indicating cortical inhibition (Nitsche and Paulus, 2000; see also, Fregni et al., 2006; Furubayashi et al., 2008; Jefferson et al., 2009; Stagg et al., 2009). Extended to the cognitive domain, it was thus assumed that improvement of a cognitive function could be achieved by anodal stimulation of the substrate underlying that function. Conversely, cathodal stimulation of the underlying substrate should lead to decrements in that function.

Several recent findings have called into question this basic premise—on which the design and interpretation of all tDCS studies hinge—thereby creating a wave of confusion. Of most pressing importance for the future of brain stimulation research, the effects of tDCS are demonstrably sensitive to seemingly subtle variations in task, stimulation parameters, and characteristics of the individuals being tested. In one well-known example from the motor domain, Batsikadze et al. (2013) charted the effects of stimulation intensity (1 mA vs. 2 mA) for both A- and C-tDCS of primary motor cortex. A-tDCS at the highest stimulation intensity produced an increase in MEPs while A-tDCS at 1 mA produced no significant change relative to baseline amplitude. Unexpectedly, C-tDCS at 2 mA *also* induced an excitatory effect (compared to the suppressed MEPs observed for C-tDCS at the level of 1 mA). Thus, the excitatory and inhibitory effects of tDCS may depend not only on the polarity of current, but also on the intensity level of stimulation. In accordance with these findings, a number of meta-analyses and review articles offer in-depth discussions of other sources of variation associated with tDCS both within and outside the motor domain (Nitsche et al., 2008; Jacobson et al., 2012; Filmer et al., 2014; Horvath et al., 2014; Li et al., 2015; Price et al., 2015). These factors include: electrode position and size (e.g., Bikson et al., 2010), timing of task relative to stimulation period (e.g., Nozari et al., 2014), duration of stimulation (e.g., Nitsche and Paulus, 2001), cognitive demand involved in task (e.g., Antal et al., 2007; Gill et al., 2015), skull thickness and subcutaneous fat content (Datta et al., 2012), and a genetic polymorphism associated with prefrontal dopamine (Plewnia et al., 2013; Nieratschker et al., 2015).

In acknowledging the challenges faced by the field of tDCS research, our aim is not to encourage the abandonment of this tool but rather to stress the value of more comprehensive experimental approach. We propose that a thorough exploration of the stimulation parameter space is essential to facilitating cross-study comparison and ultimately working toward a reliable model of tDCS effects. These steps are especially crucial for tDCS investigations in the cognitive domain, for which, relative to the motor domain, there exists greater variability in experimental design and potentially greater complexity in the neural systems engaged at task. Below, we highlight the extent of methodological variation within one sub-field of cognitive tDCS research (cognitive control), thereby motivating our own experimental approach.

Broadly construed, *cognitive control* underlies our capacity to interact flexibly with our surroundings in a goal-directed manner. More precisely, this term refers to processes such as the selection and maintenance of relevant information, shifting between tasks, and the inhibition of prepotent responses (Miller and Cohen, 2001). A combination of lesion and functional neuroimaging studies have implicated a network of cortical and subcortical brain regions as the seat of these essential functions. In particular, a host of tDCS studies have stimulated prefrontal cortex in order to affect performance on tasks of working memory, set-shifting and inhibitory control. Experimenters have examined these processes via different stimulation sites (e.g., F7- contralateral mastoid placement: Nozari et al., 2014; Fz- left cheek: Hsu et al., 2011; F3- right supraorbital (RSO): Ohn et al., 2008; the crossing point between T3-Fz and F7-Cz-RSO: Cattaneo et al., 2011; the crossing point between T4-Fz and F8-Cz: Ditye et al., 2012), and at different current intensities (e.g., 1 mA: Fregni et al., 2005; 1.5 mA: Nozari et al., 2014; 2.0 mA: Vanderhasselt et al., 2013). On top of these differences, a recent review of polarity effects on executive function (Jacobson et al., 2012) found that only half of the tDCS studies surveyed examined both cathodal *and* anodal effects. In some cases, experimenters did not include a sham stimulation condition as a control (e.g., Ditye et al., 2012). While studies generally show a boost in cognitive control during A-tDCS administered to the prefrontal cortex, it is unclear whether an equal and opposite effect would be obtained during C-tDCS under otherwise identical experimental conditions. Moreover, experimenters rarely probe dose-dependent changes in stimulation intensity (but see, Iyer et al., 2005; Hoy et al., 2013; Horvath et al., 2016), leaving open the question of whether behavioral effects might change, such as by flipping directions or diminishing across intensity levels (as in Batsikadze et al., 2013).

Against this backdrop, in which we have an abundance of data but great variation in how those data were obtained, the field is thus faced with a host of interpretation issues. To illustrate: Hoy et al. (2013) reported that anodal stimulation of left prefrontal cortex significantly improved participants’ speed on a simple work memory task (the “2-back”), but not on a more difficult version of the task (the “3-back”). They did not test the effects of cathodal stimulation. In contrast, Fregni et al. (2005) reported improved accuracy but not speed on 3-back task performed during anodal stimulation. They also tested the effects of cathodal stimulation, but found no significant effect. Finally, Zaehle et al. (2011) reported polarity-*dependent* changes in accuracy on a 2-back task (accuracy was superior for A-tDCS relative to C-tDCS), but polarity-*independent* changes in RT (reaction time was equally facilitated for A-and C-tDCS relative to sham). Though these three studies are, in fact, more closely related than is typical (i.e., they made use of the F3 electrode montage and comparable current intensity), their remaining dissimilarities still make it challenging to pinpoint the source of non-overlapping results. Can they be traced to important differences in task structure, timing of stimulation, duration of stimulation, current polarity, or some combination of these factors? In an era when replicability is increasingly a focus in psychological research (Open Science Collaboration, 2015; Anderson et al., 2016), careful consideration of these features is critical.

The present study represents a crucial step toward disentangling two basic but still crudely understood stimulation parameters within the domain of cognitive research: current polarity and stimulation intensity. While we propose the systematic variation of stimulation parameters constitutes an important contribution to the field, we fully acknowledge the limitations of this approach. Specifically, between-subject manipulations, which are useful in minimizing stimulation timing and task familiarity effects, may require prohibitively large sample sizes. Indeed, this limitation becomes even more essential when considering that the behavioral effects of tDCS may be quite small (Minarik et al., 2016).

Bearing in mind this trade-off between a comprehensive approach and lowered statistical power, we examine here performance on the Eriksen Flanker, a cognitive control task that taps into the capacity for selective attention and response inhibition (Eriksen and Eriksen, 1974), and one whose rapid pace enables us to collect a rich data set (over 550 trials). As this task has been associated with activation in prefrontal cortex (e.g., Casey et al., 2000; Ullsperger and von Cramon, 2001; Bunge et al., 2002), we selected an electrode montage thought to target prefrontal cortex in the left hemisphere: F3- RSO. In particular, we chose this montage due to its common use in studies of tDCS during various cognitive control tasks (Fregni et al., 2005; Ohn et al., 2008; Hoy et al., 2013). While the Flanker task has also been employed in a handful of other tDCS studies (Weiss and Lavidor, 2012; Nozari et al., 2014; Zmigrod et al., 2016), our design enables us to examine both the effects of current polarity (i.e., A-tDCS and C-tDCS relative to sham stimulation) and dose-dependent stimulation (i.e., to ask whether cognitive control increases or decreases monotonically from 1, to 1.5, to 2 mA). Specifically, we test the dual hypotheses that A-tDCS will improve performance on the Eriksen Flanker in a dose-dependent manner while C-tDCS will worsen performance in this way. To this end, we extract an index of cognitive control by comparing, for each participant, reaction times for trials that require response inhibition relative to those that do not strongly engage this process. By charting the parameter space of tDCS during this task, we hope to open the door to further methodological research while also serving as a launching pad for exciting theoretical questions about the behavioral consequences of suppressing and exciting cognitive control capacities.

## Materials and Methods

### Participants

One hundred and one participants recruited from the University of Pennsylvania community completed the study in exchange for $20. All were right-handed, native speakers of English between the ages of 18 and 30. They were approximately matched for education level (at minimum, all completed secondary schooling). Participants were not pregnant or currently taking psychotropic/anticonvulsive drugs. They reported no history of head trauma, seizures, or neurologic or psychiatric disease. All participants gave informed consent in accordance with the University of Pennsylvania Institutional Review Board. Of the original 101 participants who completed the study, 11 of them achieved an accuracy score less than chance performance (50%) on one or more trial types (C-tDCS: *n* = 2; S-tDCS: *n* = 4; A-tDCS: *n* = 5). As this performance suggests failure to follow task instructions, they were excluded. All analyses reported below examine the remaining 90 participants (10 per group). Corresponding demographic information is provided in **Table 1**. Though not precisely matched, sex ratios did not differ significantly by polarity manipulation (A-tDCS vs. S-tDCS: χ2 = 0.28, *p* = 0.60; C-tDCS vs. S-tDCS: χ2 = 0.08, *p* = 0.79). Furthermore, the inclusion of sex as a predictor in statistical models comparing polarity groups did not impact the pattern of significant results reported below.

**Table 1:** Summary of age and sex across experimental conditions.

Polarity	Intensity (mA)	Mean age *(SD)*	# Female
Anodal	1	21.0 (3.6)	6
Anodal	1.5	20.8 (1.9)	6
Anodal	2	21.7 (3.1)	7
Sham	1	21.1 (2.1)	3
Sham	1.5	21.2 (2.7)	9
Sham	2	20.2 (2.5)	5
Cathodal	1	22.3 (3.5)	6
Cathodal	1.5	21.5 (2.6)	6
Cathodal	2	22.6 (4.2)	6

### Stimuli

**FIGURE 1:**
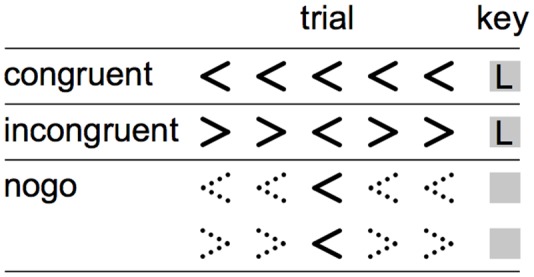
**Visualization of trials types in the Eriksen Flanker.** During the congruent and incongruent trials (*top and middle rows*), participants responded to the direction of the middle arrow. During the nogo trials, signaled by dashed flanker arrows (*bottom rows*), participants were instructed to suppress any button press.

Participants performed a nonlinguistic version of the Flanker task described in Nozari et al. (2014). An equal number (*n* = 188) of *congruent, incongruent*, and *no-go* trials were presented centrally as black text on a white background and measured approximately 0.5° × 4.5° (**Figure 1**). Each trial contained a row of five angle brackets. In *congruent* trials, the center bracket and the four flanking brackets faced the same direction (equal number of < < < < < and > > > > >). In *incongruent* trials, the center bracket and the four flanking brackets faced the opposite direction (equal number of > > < > > and < < > < <). For both *congruent* and *incongruent* trials, participants were instructed to press the left or right arrow keys to indicate the direction in which the center bracket was facing. In *no-go* trials, the four flanking brackets were constructed from dashed rather than solid lines; the bracket orientations were equally distributed between the four patterns used for *congruent* and *incongruent* trials. For these trials, participants were instructed not to make any key response. Each trial was displayed for 800 ms, followed by a fixation cross with a variable ISI drawn from a uniform distribution (500–150 ms). Trial order was randomized. Participant responses were indicated by pressing the left and right arrow keys with two fingers on the dominant hand and were recorded during the entire trial and fixation period.

### Procedure

We randomly assigned participants to one of nine between-subject experimental manipulations: three stimulation conditions (anode, sham, cathode) crossed with three stimulation intensity levels (1.0, 1.5, 2.0 mA). We varied stimulation intensity within the control groups in order to rule out the (admittedly unlikely) possibility that participants in the sham condition might, even after only 30 s of stimulation, be sensitive to differences along this dimension.

**FIGURE 2:**
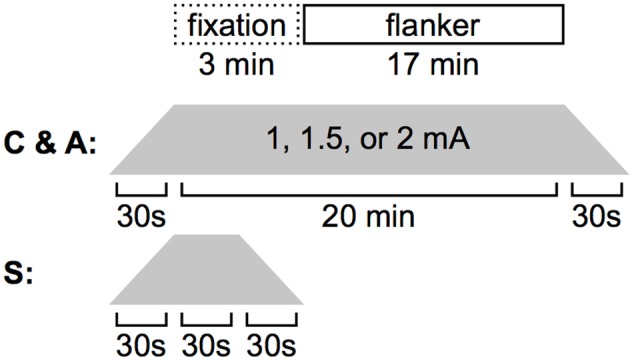
**Stimulation timing.** During A- or C-tDCS, 3 min elapsed before the Flanker task was initiated. Both stimulation and the behavioral task were terminated after 17 min. In the sham condition, current was ramped up, held steady, and ramped down in 30 s increments.

Participants were blind to their assigned condition. Experimental procedures were identical across participants (**Figure 2**). First, the experimenter applied the electrodes. Next, the experiment script was initiated; participants were informed of the task format and directed to make their responses as quickly and as accurately as possible. Once they finished reading the instruction screen (indicated by hitting the space bar), the experimenter began stimulation. The Flanker task began after an initial fixation period of 3 minutes during which the participant sat quietly. Stimulation was delivered through 5 cm × 5 cm (25 cm^2^) electrodes, placed in saline-soaked sponges and held on the head with a rubber strap. A continuous current of 1.0, 1.5, or 2.0 mA, depending on experimental condition, was generated with battery-operated continuous current stimulator (Magstim Eldith 1 Channel DC Stimulator Plus, Magstim Company Ltd., Whitland, Wales). In cathode and sham stimulation conditions, the cathode was placed over the left dorsolateral prefrontal cortex, F3 using the International 10–20 System, and the anode was placed over the right supraorbital sinus. Electrode placement was reversed for the anodal stimulation condition. In the non-sham conditions, current was increased to the target level over 30 s, held constant for 20 min (the entirety of the Flanker task + 3 min of initial fixation), and decreased to 0 over 30 s. In the sham condition, current was increased to the target level over 30 s, held constant for only 30 s, decreased to 0 over 30 s, and was maintained at 0 for the remaining 19 min 30 s. Approximately 10 min after the termination of the Flanker task, participants completed a written questionnaire in which they were asked to rate on a scale of 1–10 the extent to which they experienced the following physical sensations during the task: tingling, itching, burning, pain, headache, and change in vision.

### Analyses

**FIGURE 3:**
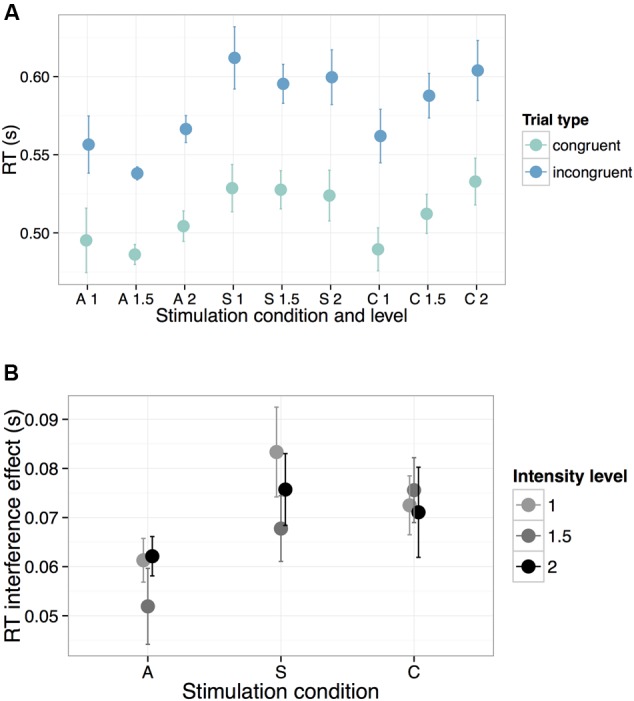
**Behavioral performance on the Flanker task across all experimental manipulations (A = Anode, S = Sham, C = Cathode).** To illustrate the general speed-up in RTs during A-tDCS, we show in **(A)** response times across all stimulation parameters for both congruent (teal) and incongruent (blue) trials. In **(B)**, we highlight the reduced interference effect during A-tDCS, plotting the mathematical difference in RTs between the incongruent and congruent trials across all levels of intensity. Error bars represent one standard error of the mean.

In preparation for analysis, we removed the first 10 trials from each participant to minimize task start-up effects (data loss 1.8%). Motivated by prior literature (e.g., Nozari et al., 2014), this pre-determined step ensured that results would not be driven by initial reaction times (RTs), which are likely to be heavily influenced by acclimation to task structure. We also excluded RTs less than 200 ms (data loss 0.04%). All results reported below hold without these trial exclusions (removal of the first 10 trials and RTs < 200 ms). Due to near-ceiling effects on accuracy on the Flanker task (mean performance = 96.7%, SE = 0.5), all subsequent analyses were carried out using reaction time (RT) on correct trials as the dependent measure (**Figure 3**).

We next implemented a linear mixed effects model (LMM) using the *lmer()* function (library *lme4*, v. 1.1–7; (Bates et al., 2014) in R (v. 3.2.2; R Development Core Team, 2015). LMMs represent a powerful, flexible tool for better estimating the generalizability of experimental findings to the broader population. Their strength lies in their ability to properly handle correlated observations (i.e., the fact that RTs for congruent and incongruent trials, collected for each subject, are necessarily non-independent) while also explicitly accounting for inter-individual variation alongside primary effects of interest. In the statistical models presented below, for example, we can evaluate the significance of our predictors of interest (stimulation condition, intensity level, and trial type) while also adding a random effects term that accounts for the possibility that, regardless of experimental manipulation, participants will be generally slower or show a smaller interference effect than others. This approach thus ensures that our observed pattern of results, particularly given our relatively small sample size, cannot be solely attributed to random variations in the sample we tested. Relative to traditional analyses of variance, LMMs are also more robust to unbalanced or missing data points and violations of compound symmetry (for detailed discussion of LMMs see, e.g., Gelman and Hill, 2006; Baayen et al., 2008; Magezi, 2015).

In light of the right skew of the RT data (skewness = 1.63), RTs from all 90 participants were first log-transformed, then regressed onto all main effects and interactions of stimulation condition (anode, sham, cathode), intensity level (1.0, 1.5, 2.0 mA) and trial type (congruent vs. incongruent). All results reported below hold without this transformation. The model also included the fullest random effects structure that allowed the model to converge: a random intercept for participant and a by-participant random slope for trial type. This random effect structure enabled us to account for inter-individual variation in overall speed of RT as well as magnitude of the interference effect. Predictors were contrast coded with a zero mean in order to reduce multicollinearity between fixed effects (*rs* < 0.6). Specifically, the condition predictor was simple coded so as to compare cathode vs. sham stimulation and anode vs. sham stimulation and the intensity predictor was reverse-helmert coded to reflect the *a priori* hypothesis that the effect of stimulation intensity would increase across levels. Because the trial type predictor includes only two levels (congruent vs. incongruent), they were directly compared to one another. All models were fit using a Restricted Maximum Likelihood procedure, which has been shown to yield unbiased variance estimates. Finally, as no-go trials required the suppression of a motor response, they could not be included in subsequent analyses.

## Results

### Examining the Effects of Current Polarity and Intensity on Flanker Performance

Results are summarized in **Table 2**. For the anode relative to the sham contrast, we found a significant main effect of condition (β = -0.069, *t* = -3.37, *p* = 0.001): overall RTs were faster during A-tDCS, regardless of trial type. Unsurprisingly, we also obtained a significant main effect of trial type: RTs were faster for the congruent relative to the incongruent trials (β = -0.063, *t* = -31.85, *p* < 0.0001), regardless of stimulation condition. Crucially, we found only one significant interaction: for anode relative to sham stimulation, the effect of trial type was reduced (β = 0.010, *t* = 2.10, *p* = 0.04). In other words, the RT penalty associated with incongruent trials was smaller during anodal stimulation (i.e., there was a smaller interference effect, **Figure 3**). As further highlighted by a simple effects analysis, the effect of trial type in the sham condition (β = -0.067, *t* = -19.45, *p* < 0.0001) was of a greater magnitude than for the A-tDCS condition (β = -0.057, *t* = -16.52, *p* < 0.0001). No reliable effect of stimulation intensity was observed, and we found no evidence that Flanker RT performance was impeded by cathodal stimulation.

**Table 2:** Coefficients (and corresponding *t*-values and *p*-values) for each predictor in a model examining the effect of stimulation condition (anode, sham, and cathode), intensity level (1–2 mA), and trial type (congruent vs. incongruent) on log-transformed RTs from the Eriksen Flanker.

Predictor	Coefficient	*T*-value	*P*-value
Condition (C vs. S)	–0.027	–1.35	0.18
**Condition (A vs. S)**	**–0.069**	**–3.37**	**0.001**
Level (1.5 vs. 1)	0.002	0.16	0.88
Level (2 vs. 1/1.5)	0.008	1.42	0.16
**Trial type (con vs. incon)**	**–0.063**	**–31.85**	**<0.0001**
Condition (C vs. S) ^∗^Level (1.5 vs. 1)	0.028	1.14	0.26
Condition (A vs. S)^∗^Level (1.5 vs. 1)	–0.005	–0.18	0.85
Condition (C vs. S) ^∗^Level (2 vs. 1/1.5)	0.021	1.44	0.15
Condition (A vs. S) ^∗^Level (2 vs. 1/1.5)	0.014	0.95	0.35
Condition (C vs. S)^∗^Trial type	0.001	0.19	0.85
**Condition (A vs. S)^∗^Trial type**	**0.010**	**2.10**	**0.04**
Level (C vs. S)^∗^Trial type	0.003	1.32	0.19
Level (A vs. S) ^∗^Trial type	0.0002	0.15	0.89
Condition (C vs. S) Level (1.5 vs. 1)^∗^ Trial type	–0.006	–0.95	0.35
Condition (A vs. S)^∗^Level (1.5 vs. 1)^∗^ Trial type	–0.002	–0.37	0.72
Condition (C vs. S) Level (2 vs. 1/1.5)^∗^ Trial type	0.003	0.79	0.43
Condition (A vs. S) Level (2 vs. 1/1.5)^∗^ Trial type	–0.00004	–0.01	0.99

*Significant values (determined using the Sattherwaite approximation and corresponding to *p* < 0.05) are bolded.*

However, inspection of **Figure 3** (top panel) revealed a striking qualitative pattern: an apparent linear increase in the effect of intensity level on RTs for the cathodal condition, independent of trial type. To probe the significance of this trend *post hoc*, intensity level was contrast-coded to test for a linear effect on the response variable within the cathodal condition. Similar to the full model detailed above, log-transformed RTs from the C-tDCS condition were regressed onto all main effects and interactions of intensity level and trial type, including a random intercept for participant and a by-participant random slope for the latter. Results revealed a significant linear increase in RTs associated with stimulation intensity (β = 0.053, *t* = 2.06, *p* = 0.049). Importantly, this trend did not differ between trial types (i.e., the interaction between intensity and trial type was not significant: β = 0.004, *t* = 0.73, *p* = 0.47). Thus, while the intensity of current for the cathodal condition had dose-dependent effects on *general* motor response time, this parameter had no unique effect on cognitive control performance.

### Examining the Effects of Physical Sensation on Flanker Performance

While no participant reported explicit awareness of the stimulation condition, we investigated whether the physical sensations experienced by participants might differ by current polarity (anode, sham, cathode). From the debriefing questionnaire, we calculated an average rating of physical sensation per participant and submitted these scores to a Mann–Whitney *U* test. Data from the debriefing questionnaire were not obtained for 8 of the 90 participants (S-tDCS: *n* = 2; C-tDCS: *n* = 3; A-tDCS: *n* = 3). Although it is widely reported that participants cannot distinguish active stimulation from sham (e.g., in double-blind sham controlled studies; Gandiga et al., 2006), our analyses revealed that anodal stimulation (mean rating = 2.88, *SE* = 0.26) was experienced differently from sham (mean rating = 1.60, *SE* = 0.25; Mann–Whitney *U* test: Z = 3.34, *p* = 0.0008): No such difference was found for cathodal stimulation (mean rating = 1.99, *SE* = 0.28) relative to sham (*Z* = 1.05, *p* = 0.29).

In light of this finding, we next examined whether performance on the Flanker task might be affected by individual differences in the strength of physical sensation experienced by the participants. If a significant effect of physical sensation was observed, particularly an interaction between physical sensation and trial type, then any observed variations in RT could be attributed to participants’ experience of A-tDCS, not necessarily to changes in cortical excitability. Using the same random effects structure described above, log-transformed RTs from the remaining 82 participants were regressed onto all main effects and interactions of stimulation condition (anode, sham, cathode), intensity level (1.0, 1.5, 2.0 mA), trial type (congruent vs. incongruent), and physical sensation ratings, mean-centered across participants. Crucially, we observed no significant main effect of physical sensation (β = -0.008, *t* = -1.23, *p* = 0.22) nor any significant interactions involving this predictor. Moreover, the original main effects of condition (anode vs. sham: β = -0.073, *t* = -2.98, *p* = 0.004) and trial type (congruent vs. incongruent: β = -0.064, *t* = -26.98, *p* < 0.0001) were maintained. Notably, the interaction between stimulation condition (anode vs. sham) and trial type was rendered marginally significant (β = 0.009, *t* = 1.49, *p* = 0.14), suggesting that some of the variance associated with that interaction was shared with the physical sensation predictor. Without the sensation predictor, we maintained the pattern of significant results described in the original model, even with the reduced number of participants (82 vs. 90).

## Discussion

Here, we systematically probed the effects of current polarity and stimulation intensity on participants’ ability to perform a task of inhibitory cognitive control. Results indicated nearly at-ceiling levels of accuracy on the Eriksen Flanker across stimulation parameters. Statistical modeling of RT data clearly showed an effect of current polarity for the anodal condition relative to sham (i.e., an overall greater speed up of RTs). Most compellingly, we have demonstrated that A-tDCS to left prefrontal cortex (via the F3-RSO electrode montage) facilitated the deployment of cognitive control resources when applied concurrently with task. This evidence was clear from the reduced difference in RTs between congruent and incongruent trials (i.e., a smaller interference effect).

Nonetheless, it is important to note that we observed significant differences in the physical sensations experienced during anodal and sham stimulation. This finding runs contrary to the bulk of the tDCS literature, which overwhelmingly reports no difference in physical perception between stimulation conditions. While it is possible that differences in physical sensation, not cortical excitability, account for observed effects in behavior, including this variable in our statistical models did not on the whole dramatically alter our results. We suggest here that participants experiencing A-tDCS may have reported increased sensitivity to stimulation because they were devoting fewer cognitive resources to perform the task required. A related possibility is that overall enhanced attentional capacity during A-tDCS may have induced learners to attend more to their physical environment. Indeed, challenging cognitive control tasks have been shown to attenuate pain intensity (Bantick et al., 2002; Valet et al., 2004; Buhle and Wager, 2010). Consonant with our findings, pain reduction in one study was shown in low working memory capacity but not high working memory capacity individuals, suggesting that attenuation of pain scales with individual differences in cognitive control (Nakae et al., 2013).

While it is best to be cautious in interpreting null effects, it is also useful to review the experimental manipulations that showed *no* effect on cognitive control. First, we observed no significant interaction between stimulation intensity (1–2 mA) and trial type (congruent vs. incongruent). In other words, cognitive control capacities were not influenced in a dose-dependent manner. A *post hoc* analysis did reveal a dose-dependent increase in *overall* RTs for the cathodal condition, but this trend did not apply to the RT difference between trial types. Most strikingly, we found no evidence that cathodal stimulation differed reliably from sham, either in significantly improving or impeding Flanker performance. While such results suggest weaker reliability of cathodal stimulation, we stress that the relatively small sample size of the current study precludes us from making definitive claims about its efficacy (Minarik et al., 2016).

Nonetheless, previous studies contextualize this null finding. Specifically, Zmigrod et al. (2016) demonstrated a significantly increased Flanker interference effect (reduced cognitive control) during cathodal stimulation of *right* prefrontal cortex (electrode montage: F4-RSO; current intensity: 2 mA), but no such effect on Simon task performance (demonstrating specificity of right PFC to stimulus-stimulus rather than stimulus–response conflict). Similar to the present findings, Nozari et al. (2014) found no evidence from either accuracy or RT measures that C-tDCS to left prefrontal cortex mediated the strength of the Flanker interference effect (electrode montage: F7-right mastoid; current intensity: 1.5 mA). Thus, it appears that cathodal stimulation of right but not left DLPFC stimulation may impede Flanker performance, but precisely why this behavioral modulation is specific to these particular stimulation conditions (C-tDCS to the right hemisphere) remains an open question. One possibility is that the Flanker task more strongly recruits right relative to left prefrontal cortex (Hazeltine et al., 2000). Indeed, recent evidence suggests that patients with right prefrontal damage showed greater Flanker interference effects than those with similar damage in the left hemisphere (Geddes et al., 2014). However, other neuroimaging studies point to more diffuse, bilateral prefrontal involvement during the Flanker (Ullsperger and von Cramon, 2001; Bunge et al., 2002; Durston et al., 2003), suggesting that the left hemisphere does assume a processing burden during tasks of inhibitory control.

In general, our findings concur with the observation that, within the broader cognitive domain, the consequences of cathodal stimulation are more varied compared to anodal stimulation (Jacobson et al., 2012). It is worth stressing that this general pattern might be traced to the observation that high-level cognitive tasks are likely to engage diffuse swathes of the brain. For example (and as indicated above), fMRI recordings of brain activation during the Flanker task have implicated widespread frontal and posterior parietal regions that are often bilaterally distributed (e.g., the middle frontal gyrus, the precentral gyrus, inferior frontal gyrus, precuneus, superior parietal lobule, etc., Zhu et al., 2010). Thus, while a stimulation-induced increase in activity in one of these areas might be enough to improve behavioral performance, suppression of an area might have unpredictable effects (likely due to compensatory recruitment of other regions). Second, whereas both A-tDCS and C-tDCS involve NMDA-receptor mediated effects, anodal stimulation effects also require sodium channel function in motor cortex (Liebetanz et al., 2002). The extent to which receptor mediated effects generalize to cognition remain to be seen. Genetically mediated individual differences in response to C-tDCS may offer a third explanation for the unreliable effects of C-tDCS. Nieratschker et al. (2015) showed that a genetic polymorphism associated with prefrontal dopamine levels predicted individual differences in behavioral response to C-tDCS. Specifically, cathodal stimulation was found to reduce cognitive control abilities in COMT 166 Val–Val homozygotes but not in Met-allele carriers (Nieratschker et al., 2015). To be clear, Plewnia et al. (2013) also showed that Met–Met homozygotes under anodal stimulation were impaired in their set-shifting abilities; however, a smaller percentage of the population are Met-allele carriers (Auton et al., 2015). Pinpointing the underlying sources of varied responses to C-tDCS, including relevant genetic determinants, is an exciting and imperative area of future research.

In sum, we have begun to disentangle the contribution of two key stimulation parameters: current polarity and intensity. Using a behavioral task that demanded the suppression of prepotent responses, we offer convincing evidence that the former, current polarity, is a robust predictor of inhibitory control abilities but that the latter, current intensity, is not. Intriguingly, this effect was specific to anodal stimulation of left prefrontal cortex, which induced a boost in cognitive control. Thus, our initial hypotheses were only partially confirmed: current polarity indeed influenced performance on a task of cognitive control. However, contrary to our expectations, this effect was specific to the anodal stimulation condition and RT interference effects did not unfold in a dose-dependent manner.

While our efforts to probe the stimulation parameter space represent an important step forward, we stress that the present approach was by no means exhaustive. Nonetheless, by providing a template for how the stimulation parameter space *might* be mapped, we open up the possibility for future research to build substantially on the findings reported here. Particularly in light of the null effects observed during cathodal stimulation, one clear next step is to examine whether these results would be overturned with a much larger sample size (i.e., perhaps C-tDCS of prefrontal cortex has a weaker, but significant behavioral impact). One might also ask whether current polarity is influenced by stimulation timing or precise electrode placement. Through increased understanding of the impact of parameter selection, a more consistent picture may emerge across cognitive tasks, and only then will truly generalizable inferences be forthcoming.

## Ethics Statement

This study was carried out in accordance with the recommendations of the University of Pennsylvania Institutional Review Board (IRB) with written informed consent from all subjects. All subjects gave written informed consent in accordance with the Declaration of Helsinki. The protocol was approved by the IRB.

## Author Contributions

EK, ZB, RH, and ST-S formulated the experiment. EK and ZB implemented the experiment, supervised data collection and performed data analysis. RH, JM, NT, and ST-S provided input on stimulus materials and data analysis. EK and ZB wrote the original draft of the paper, with revisions and additional content contributions from RH, JM, NT, and ST-S.

## Conflict of Interest Statement

The authors declare that the research was conducted in the absence of any commercial or financial relationships that could be construed as a potential conflict of interest.
